# Factors associated with adherence to post-exposure prophylaxis among dental professionals in Brazil

**DOI:** 10.1590/1807-3107bor-2025.vol39.107

**Published:** 2025-10-10

**Authors:** Gustavo Correia Basto da SILVA, Ana Carolina Marques Medeiros VIANI, Angélica Maria Cupertino Lopes MARINHO, Isabela Almeida PORDEUS, Mauro Henrique Nogueira Guimarães de ABREU

**Affiliations:** (a) Universidade Federal de Minas Gerais – UFMG, School of Dentristry, Postgraduate Program in Dentistry, Belo Horizonte, MG, Brazil.; (b) Universidade Federal de Minas Gerais – UFMG, School of Dentristry, Department of Oral Health for Children and Adolescents, Belo Horizonte, MG, Brazil.; (c) Universidade Federal de Minas Gerais – UFMG, School of Dentristry, Department of Social and Preventive Dentistry, Belo Horizonte, MG, Brazil.

**Keywords:** Disease Transmission, Infectious, Post-Exposure Prophylaxis, Dentistry, Epidemiological Monitoring, Guideline Adherence

## Abstract

This study analyzed the influence of sociodemographic, occupational, and accident-related factors on adherence to post-exposure prophylaxis (PEP) among dental professionals. A cross-sectional study was conducted using secondary data from the Brazilian Notifiable Diseases Information System, covering incidents of biological material exposure from 2018 to 2023 across all federal units. PEP adherence was the outcome analyzed, and related factors included sociodemographic, occupational, and accident characteristics. Descriptive, bivariate, and multivariate logistic regression analyses were performed (α = 5%). A total of 15,650 notifications involving dental professionals were analyzed, representing 3.91% of all cases of occupational exposure, with a PEP adherence rate of 91.0%. The exposed professionals had a mean age of 32.5 years (SD = 10.4), were predominantly women (76.6%), of White race/ethnicity (65.4%), and had higher education (79.7%). The mean work experience was 3.6 years (SD = 6.8). In the adjusted model, adherence was associated with lower educational attainment (OR = 0.71; 95%CI: 0.58–0.88), younger age (OR = 0.99; 95%CI: 0.98-0.99), and longer work experience (OR = 1.02; 95%CI: 1.01-1.03). Percutaneous exposure (OR = 1.39; 95%CI: 1.16–1.67), contact with blood (OR = 1.47; 95%CI: 1.18–1.82), and dental procedure-related accidents (OR = 1.35; 95%CI: 1.07–1.70) increased the likelihood of adherence. Hepatitis B vaccination (OR = 1.96; 95%CI: 1.62–2.36) was associated with increased adherence to PEP. Adherence to PEP was influenced by multiple factors, highlighting the need for effective strategies to improve adherence and occupational safety among these professionals.

## Introduction

Occupational accidents with exposure to biological material (ATMB) represent a global public health issue, and involve the exposure of workers to potentially contaminated fluids through percutaneous or mucosal injuries, thereby increasing the risk of transmission of infectious diseases. Among healthcare professionals, percutaneous injuries are a leading form of occupational exposure, commonly linked to pathogens such as hepatitis B (HBV), hepatitis C (HCV), and human immunodeficiency virus (HIV).^1,2^ Studies report a high prevalence of occupational exposures in dental settings, as these professionals are at increased risk due to direct contact with patients’ oral cavities, frequent use of sharp instruments, and handling of high-speed rotary devices that produce potentially contaminated aerosol.^3,4^ Up to 63.05% of dental professionals reported injuries during clinical practice.^5^

The Centers for Disease Control and Prevention (CDC) recommends that all healthcare professionals adopt standard precautions (SP), defined as a set of measures to reduce the risk of infection transmission during patient care, regardless of diagnosis.^2^ In Brazil, the National Health Surveillance Agency (ANVISA) reinforces these guidelines, and include hand hygiene, use of personal protective equipment (PPE), immunization, and proper disposal of sharp instruments.^6^ However, the adherence to these measures is often inadequate^4^. Studies indicate that although many dentists report following prophylactic measures, adherence to SP in post-exposure protocols varies widely, with rates ranging from 4.5% to 83.9%.^4,7^

The use of PPE reduces contact between potentially contaminated biological materials and skin or mucous membranes but does not consistently prevent injuries from sharp instruments.^4^ Professionals exposed to such materials should promptly seek reference services for evaluation, potential post-exposure prophylaxis (PEP), and periodic counseling and follow-up.^6^ PEP involves antiretroviral medication to reduce infection risk following a high-risk exposure.^6^ In case of occupational exposure, PEP is indicated when the serology of the source patient is positive, inconclusive, or unavailable.^8^ Evidence shows that timely use of PEP can reduce the risk of infection among healthcare professionals by up to 81%.^9,10^ In Brazil, the clinical protocol for PEP is regularly updated, and the 2024 revision introduced new recommendations and reinforced guidance, detailing indication criteria and adjusting antiretroviral regimens and dosing schedules.^6^

Evidence shows that antiretroviral medications can cause adverse events, which may negatively impact the adherence of healthcare professionals to recommended protocols.^11^ Research has explored determinants of adherence.^4,8,9^ A prospective study in Ghana reported full PEP adherence with a short-term (3-day) regimen, while adherence to 28-day regimens ranged from 56% to 62%^9^. In Brazil, a study on occupational PEP adherence among dentists found a 51.5% adherence rate^4^. Despite these findings, understanding the adherence to occupational post-exposure prophylaxis remains limited,^9^ particularly in dentistry.^1^ Factors such as the profile of the injured professional, exposure context, and hepatitis B vaccination status remain underexplored, hindering improvements in prevention and management strategies for occupational exposures. Additionally, the need for studies with broader geographic coverage highlights the importance of research on this topic.

Thus, this study aimed to analyze the influence of sociodemographic, occupational, and accident-related factors on adherence to PEP among dental professionals in Brazil.

## Methods

### Ethical aspects

This study did not require prior approval from a Research Ethics Committee, as it used publicly accessible data provided by the Brazilian Ministry of Health, which did not contain identifiable information about reported cases. The research adhered to ethical guidelines for data handling, analysis, and publication, following Resolutions 466 of December 12, 2012 and 510 of April 7, 2016, from the Brazilian National Health Council, in accordance with the Declaration of Helsinki. This study was reported in accordance with the Strengthening the Reporting of Observational Studies in Epidemiology (STROBE) guidelines.

### Study design and setting

This was a population-based cross-sectional study that analyzed occupational accidents involving biological material among dental professionals. Publicly available national databases (TabWin), provided by DATASUS through the website https://datasus.saude.gov.br/transferencia-de-arquivos, were used. These databases contain records of “ACBI” notifications from SINAN-NET. The analysis covered the period from January 2018 to December 2023.

The Information System of Notifiable Health Conditions (SINAN) standardizes the collection, processing, and consolidation of data on notifiable diseases and health conditions across Brazil. It is available in all municipalities and states, it supports morbidity analysis and health intervention planning. Notifications, as defined in the National List of Notifiable Diseases,^12^ are submitted by healthcare professionals or citizens at various levels of the health system.

### Participants

All dental professionals were included according to the Brazilian Classification of Occupations (CBO), aligned with the International Standard Classification of Occupations (ISCO): dentists (and their specialties), dental assistants, dental technicians, and dental laboratory technicians. Notifications with missing data for the variables “occupation” and “refusal of indicated PEP” were excluded.

Figure is a flowchart showing the frequency of exposure to biological material among dental professionals, highlighting patient identification as a known or unknown source, an essential aspect for PEP indication according to the protocol.^6^ All possible combinations of HBsAg+, anti-HIV+, anti-HBc+, and anti-HCV+ markers are shown in absolute and relative values.

**Figure:**
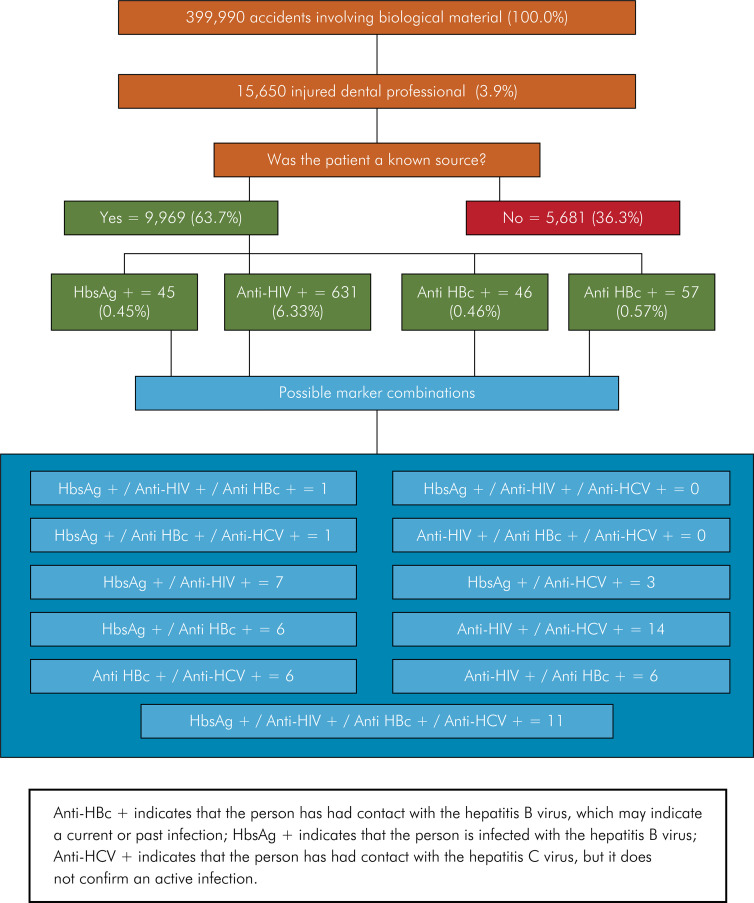
Serological profile of source patients involved in occupational accidents with exposure to biological material among dental professionals.

### Variables

The outcome variable was defined as PEP adherence, dichotomized as yes or no. Adherence to PEP occurred when the professional agreed to follow the recommended prophylaxis during post-accident care.^6^ Notifications with the outcome recorded as unknown were recoded as non-adherence. Independent variables were categorized into: a) sociodemographic, b) occupational, and c) accident-related factors.

a.Sociodemographic:

- Sex was dichotomized into male and female.
- Race/ethnicity was categorized as Black (Black and Brown), White, and others (Indigenous/Asian).
- Education was dichotomized into high school and college or university education, based on the composition of the dental workforce in Brazil. Technicians and assistants generally have a high school education, as this is a minimum requirement for this position, while dentists have at least a higher education degree^13^.
- Age was recorded in years.

b.Occupational:

- Work experience was recorded in years.
- Occupation was dichotomized into assistant/technician (dental assistant, dental technician, dental prosthesis assistant, dental prosthesis technician) and dentist^14^.
- Employment status was categorized into three groups: employer; informal employment (unregistered employee, self-employed, unemployed, temporary worker, cooperative member, casual worker, and others); and formal employment/retired (registered employee, statutory public servant, and contracted public servant).

c.Accident-related:

- Percutaneous exposure (yes/no).
- Mucocutaneous exposure (yes/no).
- Type of organic material was dichotomized into blood/blood-containing fluids and other fluids (plasma, serum, and cerebrospinal fluid).
- Type of accident was categorized into three groups: non-dental procedures (medication administration, punctures, etc.), improper disposal/handling, and dental procedures.
- PPE use was assessed through the sum of dichotomous variables of use of gloves, gown, goggles, and mask, adapted from Bertelli et al. (2023)^2^. The presence of each item was scored as 1, and absence was scored as 0. A total score of 4 indicated adequate PPE use, while scores from 1 to 3 were classified as inadequate PPE use.
- Hepatitis B vaccination status was dichotomized into yes or no.

### Statistical analysis

Descriptive analysis was performed for all variables, with categorical variables presented as absolute and relative frequencies. Quantitative variables were described using measures of central tendency and dispersion, with normality assessed by the Kolmogorov-Smirnov test with Lilliefors correction and graphical analyses such as histograms and stem-and-leaf plots.

Binary logistic regression models were developed to evaluate the association between sociodemographic, occupational, and accident-related factors and PEP adherence. Unadjusted logistic regression models were used to estimate crude odds ratios (OR) and their respective 95% confidence intervals (95%CI) for each covariate. Covariates with p-values < 0.20 in the unadjusted analysis were included in the final multiple regression model, and only variables showing p-value < 0.05 in the adjusted analysis were retained. Crude and adjusted ORs with 95% CIs were estimated.

Model adequacy was assessed using the Hosmer-Lemeshow test. Collinearity was checked through variance inflation factor (VIF) values, and Cook’s distance was used to analyze residuals. Analyses were performed using SPSS version 28.0 (SPSS for Windows, IBM Inc., Armonk, USA).

## Results

The study evaluated 399,990 notifications of incidents involving exposure to biological materials, of which 15,650 (3.91%) involved dental professionals. No notifications were recorded for the role of Dental Prosthesis Assistant. In 63.7% of the cases, patients with known or confirmable serology at the time of the incident were identified as the source. Of these, 631 tested positive for HIV, 57 for HCV, 46 for anti-HBc, and 45 for HBsAg. Eleven cases showed simultaneous positivity for HBsAg, anti-HIV, anti-HBc, and anti-HCV, while less frequent combinations included seven cases with positivity for HBsAg and anti-HIV (Figure).

The majority of professionals adhered to PEP (91.0%). The mean age was 32.5 years (SD = 10.4), with a predominance of women (76.6%), White race/ethnicity professionals (65.4%), and those with higher education (79.7%). Professionals had a mean work experience of 3.6 years (SD = 6.8). Most professionals were dentists (87.6%), with 52.8% having no formal employment contract. Most accidents were percutaneous exposure (81.8%), while mucocutaneous exposure was less frequent (8.5%). Exposure to blood or blood-containing fluids was predominant (88.0%). The most frequent type of accident involved dental procedures (76.3%). Adequate PPE use was observed in 53.9% of cases, and most professionals (84.5%) were vaccinated against hepatitis B (Table 1).

**Table 1:** Characteristics of dental professionals exposed to biological materials, Brazil (2018–2023).

Variables	n (%)
PEP Adherence	
Yes	10252 (91.0)
No	1009 (9.0)
Sociodemographic data	
Sex	
Male	3662 (23.4)
Female	11988 (76.6)
Race/Ethnicity	
Black	4981 (33.1)
White	9842 (65.4)
Indigenous/Asian	218 (1.4)
Education	
High school or equivalent	2830 (20.3)
Higher education	11120 (79.7)
Age	M 32.5 SD 10.4 Median 29.0 P25: 25.0; P75: 38.0
Occupational data	
Work experience	M 3.6 SD 6.8 Median 0.5 P25: 0.0; P75: 4.0
Occupation	
Assistant/Technician	1945 (12.4)
Dentist	13705 (87.6)
Employment status	
Employer	62 (0.5)
Informal employment	6898 (52.8)
Formal employment/retired	6103 (46.7)
Accident data	
Percutaneous exposure	
Yes	12032 (81.8)
No	2684 (18.2)
Mucocutaneous exposure	
Yes	1133 (8.5)
No	12234 (91.5)
Type of organic material	
Other fluids	1793 (12.0)
Blood/Blood-containing fluids	13134 (88.0)
Type of accident	
Non-dental procedures	1699 (11.4)
Improper disposal/Handling	1825 (12.3)
Dental procedures	11323 (76.3)
PPE use	
Inadequate PPE use	5988 (41.5)
Adequate PPE use	8430 (53.9)
Vaccination status of the injured professional for Hepatitis B	
Not vaccinated	2303 (15.5)
Vaccinated	12515 (84.5)

PEP: Post-Exposure Prophylaxis; n: absolute frequency; M: mean; P25–P75: interquartile range (IQR); SD: standard deviation; PPE: Personal Protective Equipment.

The covariates associated with PEP adherence in the unadjusted model were race/ethnicity, education, age, work experience, percutaneous exposure, mucocutaneous exposure, type of organic material, type of accident, PPE use, and hepatitis B vaccination.

In the final adjusted model, PEP adherence was associated with multiple factors. Professionals with higher education had a lower likelihood of adherence compared to those with a high school education or similar (OR = 0.71; 95%CI: 0.58–0.88). Each additional year of age slightly reduced the likelihood of adherence (OR = 0.99; 95%CI: 0.98–0.99), while longer work experience was positively associated with adherence (OR = 1.02; 95%CI: 1.01–1.03). Percutaneous exposure (OR = 1.39; 95%CI: 1.16–1.67) and contact with blood or contaminated fluids (OR = 1.47; 95%CI: 1.18–1.82) was associated with increased adherence. Accidents during dental procedures were also associated with increased adherence compared to other types of accidents (OR = 1.35; 95%CI: 1.07–1.70). Hepatitis B vaccination (OR = 1.96; 95%CI: 1.62–2.36) was associated with higher adherence to PEP (Table 2).

**Table 2:** Factors associated with PEP Adherence among dental professionals (N = 9697), Brazil (2018–2023).

Variables	Adherence n (%)	Unadjusted OR (95%CI)	p-value	Adjusted OR (95%CI)	p-value
Sociodemographic data					
Sex					
Male	2420 (91.3)	1			
Female	7832 (91.0)	0.96 (0.82–1.12)	0.611		
Race/Ethnicity					
Black	3241 (91.0)	1			
White	6589 (92.1)	1.15 (1.00–1.33)	0.051*		
Indigenous/Asian	137 (90.7)	0.97 (0.55–1.70)	0.904		
Education					
High school or equivalent	1970 (93.5)	1		1	
Higher education	7434 (91.7)	0.77 (0.64–0.93)	0.008*	0.71 (0.58–0.88)	0.001**
Age		1.00 (0.99–1.00)	0.178*	0.99 (0.98–0.99)	0.004**
Occupational data					
Work experience		1.01 (1.00–1.02)	0.039*	1.02 (1.01–1.03)	0.016**
Occupation					
Assistant/Technician	1198 (91.2)	1			
Dentist	9054 (91.0)	0.97 (0.79–1.19)	0.786		
Employment status					
Employer	47 (95.9)	1			
Informal employment	4657 (91.3)	0.44 (0.11–1.83)	0.262		
Formal employment/retired	4204 (91.6)	0.47 (0.11–1.92)	0.291		
Accident data					
Percutaneous exposure					
No	1832 (87.7)	1		1	
Yes	8358 (92.0)	1.61 (1.39–1.88)	< 0.001*	1.39 (1.16–1.67)	< 0.001**
Mucocutaneous exposure					
No	9307 (91.6)	1			
Yes	748 (89.7)	0.79 (0.63–1.00)	0.055*		
Type of organic material					
Other fluids	1108 (84.3)	1		1	
Blood/Blood-containing fluids	9071 (92.0)	2.14 (1.81–2.52)	< 0.001*	1.47 (1.18–1.82)	0.001**
Type of accident					
Non-dental procedures	1020 (85.9)	1			
Improper disposal/Handling	1258 (91.3)	1.73 (1.35–2.21)	< 0.001*	1.13 (0.83–1.53)	0.429
Dental procedures	7793 (91.7)	1.82 (1.52–2.18)	< 0.001*	1.35 (1.07–1.70)	0.010**
PPE use					
Inadequate PPE use	4316 (90.5)	1			
Adequate PPE use	5841 (91.5)	1.13 (1.00–1.29)	0.057*		
Vaccination status of the injured professional for Hepatitis B					
Not vaccinated	1341 (83.6)	1		1	
Vaccinated	8810 (92.3)	2.35 (2.02–2.73)	< 0.001*	1.96 (1.62–2.36)	< 0.001**

PEP: Post-Exposure Prophylaxis; n: absolute frequency; PPE: Personal Protective Equipment; OR: odds ratio; CI: confidence interval; *variables included in the multivariate model (p < 0.20); **variables that remained in the final multivariate model (p < 0.05).

### Model fit statistics

Data showed a non-normal distribution. The Hosmer-Lemeshow test indicated good model fit to the sample (χ^2^ = 3.078; df = 8; p = 0.929). The model was statistically significant [χ^2^(8) = 116.339; p < 0.001; Nagelkerke R^2^ = 0.033].

## Discussion

In this study, adherence to PEP was influenced by sociodemographic, occupational, and accident-related factors. Professionals with lower educational attainment, younger age, and longer work experience showed a higher likelihood of adherence. Percutaneous exposure, contact with blood or blood-containing fluids, and accidents related to dental procedures were also significantly associated with higher odds of adherence. Hepatitis B vaccination stood out as an essential protective factor. Although the model explained only a small proportion of the variance in adherence behavior, it demonstrated good fit according to the Hosmer-Lemeshow test, and all statistical assumptions were met. This suggests that the model remains valid and that additional behavioral, organizational, or contextual factors may play a role and warrant further exploration in future studies.

The rate of biological material exposure incidents observed in our study was relatively low in dental professionals. In contrast, a systematic review on the prevalence of percutaneous injuries in dentists revealed a significant variation in Brazilian studies, with rates ranging from 4.85% to 85.24%.^1^ This range reflects the specific nature of the populations studied, as the systematic review included only dentists, who use sharp instruments more frequently and, consequently, are at higher risk of biological material incidents, which may explain the differences in reported prevalence rates. Additionally, unlike the studies included in the review^1^ that relied on retrospective data susceptible to recall bias, this research minimized that limitation by using data recorded immediately after the incident or within 72 hours of its occurrence. However, it is important to highlight that the study analyzed data from a nation-wide sample composed of professionals from various fields, including not only healthcare workers but also any workers in occupations listed in the CBO that could potentially have contact with biological material. Another consideration is the potential difference in reporting rates between dental professionals in public health settings and those in private practice. Public sector professionals may be more familiar with reporting protocols and better access to occupational health services^15^. Future studies could investigate this difference by comparing data from both sectors.

Another consideration is the higher proportion of occupational accidents among female dental professionals. This finding may reflect the ongoing feminization of the dental workforce in Brazil, where women currently represent the majority of registered dentists.^16^ Similar trends have also been observed internationally, with studies showing a growing proportion of women in dentistry and distinct professional practice patterns compared to men.^17^ These demographic changes may influence exposure profiles and notification behavior, which should be explored in future research.

Testing of both the source person and the exposed individual is essential for early diagnosis and timely treatment, even in the absence of specific measures to reduce HCV infection risk after exposure.^6^ In our sample, the rate of reported cases with positive HCV source persons was 0.87%. The Brazilian Ministry of Health protocols recommend monitoring the exposed individual if the source person’s rapid test is reactive, due to the risk of seroconversion, and performing HCV viral load testing to confirm active infection and determine follow-up action.^6^.Continuous monitoring of the exposed individual and raising awareness among professionals for strict adherence to post-exposure procedures are essential.

PEP adherence was high among dental professionals in our sample, contrasting with previous studies that reported adherence rates of 51.5% among dentists in Brazil^4^, 17.9% among healthcare professionals in Ghana,^8^ and between 56% and 62% in another hospital in Ghana.^9^ These differences may be attributed to variations in adherence criteria adopted, as other studies considered measures beyond PEP^4,8^ or the completion of the prophylactic regimen.^9^ In Brazil, the higher adherence may be associated with advances in educational policies, such as the National Policy on Permanent Health Education, which strengthened the relationship between health services and educational institutions, promoting significant changes in team work processes.^18^ Advances may have included the application of safety measures in the professionals’ daily routines, as the mere transfer of knowledge during training is not sufficient to foster behavior change. In this context, it is essential to observe the problems encountered in daily work and implement strategies to change reality based on these observations.^19^ Additionally, greater access to information, training, and changes in occupational safety culture over the years may also have contributed to this outcome.

Studies indicate a higher frequency of adverse events, particularly nausea, weakness, malaise, dizziness, and psychological impact, in patients undergoing PEP with multidrug regimens compared to HIV-positive individuals using similar medications.^20,21^ The frequency adverse events is higher among patients on triple therapy than those on dual therapy. However, the preferred regimen adopted in Brazil^6,22^ (tenofovir/lamivudine + dolutegravir) has lower toxicity and a simplified dosing regimen, facilitating treatment adherence.^23^

Education showed an inverse association with PEP adherence, reflecting differences in risk perception, the influence of beliefs, or reliance on other protective mechanisms among more qualified professionals.^24^ Data indicate that professionals who perceive exposures as low risk are up to 96% less likely to adhere to the PEP protocol, while PEP training significantly increases adherence,^8^, highlighting the importance of educational initiatives. Age also showed an inverse association with PEP adherence. This finding, which is consistent with other studies,^4,8^ suggests that younger professionals may be more attentive or inclined to follow safety protocols, possibly due to recent exposure to educational content. Additionally, younger professionals may be more at risk of occupational accidents, corroborated by the National Health Survey,^25^ which found a higher likelihood of occupational accidents among younger respondents, possibly due to the fact that the labor market prioritizes professionals in this age group. On the other hand, work experience was positively associated with PEP adherence, suggesting that professionals with more years in the field may be more likely to report adherence. However, given the cross-sectional nature of the data, no causal inference can be made. Moreover, the influence of training stands out as a relevant factor, as indicated by another study where healthcare professionals who received PEP training were four times more likely to adhere to the protocol^8^. Thus, the inverse association between age and education, in contrast to the positive association of work experience, suggests that professional experience influences risk perception and adherence. While greater experience may increase awareness of occupational risks, higher age and educational attainment may lead to overconfidence or risk underestimation, impacting adherence. Nonetheless, given the limited explanatory power of the model, these associations should be interpreted with caution, as a substantial portion of the variability in adherence behavior remains unexplained.

The development of diseases after exposure to biological materials depends on the transmission potential of the infectious agent and factors such as the type of accident, nature of exposure, type of fluid involved, and serological status of both the source and the exposed individual.^9^ Among exposure types, percutaneous incidents involve injuries caused by sharp instruments, while mucosal exposures result from the contact of mucous membranes (eyes, nose, mouth, or genitals) with potentially contaminated fluids^6^. In this study, percutaneous exposure was more prevalent than mucosal exposure, aligning with previous findings,^3^ and showed a significant association with higher PEP adherence among exposed professionals compared to non-exposed ones. The low frequency of mucosal exposures may indicate greater adherence to protective equipment, possibly heightened by the COVID-19 pandemic, as the study period covered four years of this scenario. Percutaneous exposure, due to the evident risk of pathogen transmission associated with the presence of blood at the puncture site and the limited protection provided by PPE,^4^ may encourage greater adherence to safety protocols. Therefore, the importance of specific interventions to prevent percutaneous exposures, such as training on safe handling of biological material, is highlighted.

Blood is the most common biological material in occupational accidents, representing a concern due to the risk of pathogen transmission.^26^ Literature indicates that accidents involving sharp objects, such as hollow needles contaminated with blood, significantly increase the risk of seroconversion to HIV, HCV, and HBV.^27^ In the analyzed Brazilian context, blood exposure was more prevalent, consistent with other studies,^4,8^ increasing the likelihood of PEP adherence by 37% compared to exposure to other fluids. The findings highlight the importance of prioritizing the safe handling of blood-containing materials in prevention strategies, considering their potential as a medical emergency.^6^ Additionally, the amount of blood involved in the injury should be carefully considered, which is a limitation of this study due to the lack of detailed specification in national data.

The data from this study confirmed the association between accidents related to dental procedures and higher adherence to PEP, suggesting that the perceived risk by professionals may positively influence their decision to follow the protocol. The literature supports this perception, showing that both dental professionals and the general population consider dental procedures highly hazardous for infection transmission, especially during incidents involving sharp injuries.^28^ Previous studies with dental students found that syringe needles are the primary sources of accidents, followed by dental burs and suture needles.^3,29^ The characteristics of these accidents suggest that the left index finger is the most vulnerable anatomical site. Key contributing factors include lapses in concentration, followed by lack of time and technical training.^29^

Dental professionals vaccinated against hepatitis B had a higher likelihood of adhering to PEP compared to unvaccinated individuals, consistent with other findings,^4^ suggesting that knowledge of biological risks influences the adoption of such measures. Given the high HBV infection rate among dentists compared to the general population,^29^ it is plausible that these professionals are more aware of the importance of prophylaxis, reflecting a greater commitment to biosafety protocols. Additionally, how professionals understand the health-disease process and adopt an active stance on self-care may directly influence adherence to prophylactic measures.^30^

The limitations of this study must be acknowledged. The use of secondary data may introduce inconsistencies due to underreporting and variability in data quality, which can depend on the experience of the reporting professional and regional disparities in surveillance infrastructure, potentially resulting in information bias. These limitations may particularly affect the nationwide representativeness of the findings, especially in resource-limited areas where underreporting tends to be more pronounced. Moreover, the relatively low number of notifications involving dental professionals may reflect underreporting within this subgroup. It is also important to note that the database includes a wide range of occupations potentially exposed to biological material, not exclusively healthcare workers. This broader inclusion criterion may have diluted the proportion of dental professionals in the sample.

Additionally, specific details such as the volume of blood involved in the exposure and the time elapsed before PEP initiation were not recorded. Causal relationships between the analyzed variables cannot be established, requiring cautious interpretation of the findings. Furthermore, the results should be considered in light of the database structure, which assessed adherence to treatment during post-exposure care, without longitudinal follow-up to confirm treatment completion. The high adherence rate observed may reflect unique characteristics of the sample.

However, these limitations do not compromise the validity of the results, as the study stands out for using official nationwide data reported in real-time, minimizing recall bias. In addition to including variables not yet explored in the literature, the study adopted measures to minimize biases, such as selecting notifications of dental professionals based on CBO codes, reducing selection bias. Ignored variables were treated as specific categories to preserve information and minimize impacts on results. The reorganization of categorical variables avoided limitations from low frequency, and the consistent definition of the time interval prevented seasonal biases or influences from protocol changes. Therefore, future studies should adopt longitudinal designs to assess adherence to PEP and verify treatment completion among dental professionals. Specifically, prospective cohort studies sould be designed to capture key variables not available in secondary data sources, including access to PEP services, institutional problem-solving support, and psychosocial factors.

## Conclusion

PEP adherence was influenced by sociodemographic, occupational, and accident-related factors. Professionals with lower educational attainment and greater work experience demonstratedhigher adherence, while increasing age was associated with a reduced probability. Percutaneous exposure, contact with blood, and accidents during dental procedures were associated with higher adherence. Hepatitis B vaccination also favored adherence. These findings highlight the importance of more effective strategies for monitoring exposed professionals, ensuring continuous support during and after PEP, and guaranteeing uninterrupted access to stigma-free PEP services. Additionally, implementing psychological support may be essential to mitigate post-traumatic stress and strengthen occupational safety. However, due to potential underreporting and the nature of the secondary data, the findings should be interpreted with caution and are primarily generalizable to reported cases, not necessarily to all dental professionals exposed to biological material.

## Data Availability

The authors declare that all data generated or analyzed during this study are included in this published article.

## References

[1] Pereira MC, Mello FW, Ribeiro DM, Porporatti AL, Costa S, Flores-Mir C (2018). Prevalence of reported percutaneous injuries on dentists: a meta-analysis. J Dent.

[2] Bertelli C, Martins BR, Reuter CP, Krug SB (2023). Acidentes com material biológico: fatores associados ao não uso de equipamentos de proteção individual no Sul do Brasil. Cien Saude Colet.

[3] Shaghaghian S, Golkari A, Pardis S, Rezayi A (2015). Occupational exposure of shiraz dental students to patients' blood and body fluid. J Dent (Shiraz).

[4] Martins AM, Pereira RD, Ferreira RC (2010). Adesão a protocolo pós-exposição ocupacional de acidentes entre cirurgiões-dentistas. Rev Saude Publica.

[5] Savic Pavicin I, Lovric Ž, Zymber Çeshko A, Vodanovic M (2020). Occupational injuries among dentists in Croatia. Acta Stomatol Croat.

[6] Ministério da Saúde (BR) (2024). Protocolo clínico e diretrizes terapêuticas para Profilaxia Pós-Exposição de Risco (PEP) à infecção por HIV, ISTs e hepatites virais.

[7] Garcia LP, Blank VL (2006). [Prevalence of occupational exposures to potentially infectious materials among dentists and dental assistants]. Cad Saude Publica.

[8] Suglo RE, Aku FY, Anaman-Torgbor JA, Tarkang EE (2021). Predictors of adherence to HIV Post-Exposure Prophylaxis protocol among frontline healthcare workers at the Ho Teaching Hospital, Ghana. Int J Infect Dis.

[9] Tetteh RA, Nartey ET, Lartey M, Mantel-Teeuwisse AK, Leufkens HG, Nortey PA (2015). Adverse events and adherence to HIV post-exposure prophylaxis: a cohort study at the Korle-Bu Teaching Hospital in Accra, Ghana. BMC Public Health.

[10] Mabwe P, Kessy AT, Semali I (2017). Understanding the magnitude of occupational exposure to human immunodeficiency virus (HIV) and uptake of HIV post-exposure prophylaxis among healthcare workers in a rural district in Tanzania. J Hosp Infect.

[11] Nyame L, Hu Y, Xue H, Fiagbey ED, Li X, Tian Y (2024). Variation of adverse drug events in different settings in Africa: a systematic review. Eur J Med Res.

[12] Ministério da Saúde (BR) Portaria GM/MS N^o^ 5.201, de 15 de agosto de 2024. ltera o Anexo 1 do Anexo V à Portaria de Consolidação MS no 4, de 28 de setembro de 2017, para incluir novas doenças na Lista Nacional de Notificação Compulsória de doenças, agravos e eventos em saúde pública, nos serviços de saúde públicos e privados em todo o território nacional, e modifica o Anexo XLIII à Portaria de Consolidação MS no 5, de 28 de setembro de 2017, para revogar o item I da Lista Nacional de Doenças e Agravos a serem monitorados pela Estratégia de Vigilância Sentinela.

[13] Figueirêdo EC, Silva AFD, Oliveira AN, Pereira JV (2020). Categorias auxiliares em odontologia: análise e caracterização do panorama da distribuição no Brasil. Res Soc Develop.

[14] Abreu MH, Lopes-Terra MC, Braz LF, Rímulo AL, Paiva SM, Pordeus IA (2009). Attitudes and behavior of dental students concerning infection control rules: a study with a10-year interval. Braz Dent J.

[15] Trayner KM, Hopps L, Nguyen M, Christie M, Bagg J, Roy K (2018). Cross-sectional survey of a sample of UK primary care dental professionals' experiences of sharps injuries and perception of access to occupational health support. Br Dent J.

[16] Martorell LB, Silva ALM, Leles CR, Silva BSDF, Santos CVMD, Finkler M (2021). Gender differences among dentistry conference speakers in Brazil. Saude Debate.

[17] Surdu S, Mertz E, Langelier M, Moore J (2021). Dental workforce trends: a national study of gender diversity and practice patterns. Med Care Res Rev.

[18] Jesus JMD, Rodrigues W (2022). Trajetória da Política Nacional de Educação Permanente em saúde no Brasil. Trab Educ Saúde.

[19] Gomes SC, Mendonça IV, Oliveira LP, Caldas AJ (2019). Acidentes de trabalho entre profissionais da limpeza hospitalar em uma capital do Nordeste, Brasil. Cien Saude Colet.

[20] P V, Carli GD, Ippolito G (2005). Italian Registry of antiretroviral post-exposure prophylaxis. postexposure HIV prophylaxis regimen. Clin Infect Dis.

[21] Young T, Arens FJ, Kennedy GE, Laurie JW, Rutherford GW (2007). Antiretroviral post-exposure prophylaxis (PEP) for occupational HIV exposure. Cochrane Database Syst Rev.

[22] Domingues K (2016). Announcement: updated guidelines for antiretroviral postexposure prophylaxis after sexual, injection-drug use, or other nonoccupational exposure to HIV - United States, 2016. MMWR Morb Mortal Wkly Rep.

[23] Valin N, Fonquernie L, Daguenel A, Campa P, Anthony T, Guiguet M (2016). Evaluation of tolerability with the co-formulation elvitegravir, cobicistat, emtricitabine, and tenofovir disoproxil fumarate for post-HIV exposure prophylaxis. BMC Infect Dis.

[24] Auerbach JD, Malone S, Forsyth AD (2024). Occupational post-exposure prophylaxis among healthcare workers: a scoping review of factors affecting optimal utilization. J Int AIDS Soc.

[25] Gomides LD, Abreu MN, Assunção AÁ (2024). Desigualdades ocupacionais e diferenças de gênero: acidentes de trabalho, Brasil, 2019. Rev Saude Publica.

[26] Younai FS (1996). Postexposure protocol. Dent Clin North Am.

[27] Bestaoui S, Amar NH, Bouhalloufa HF (2024). Accidental exposure to blood (AEB), a real public health problem for medical and paramedical staff at the university hospital of Mostaganem. Int J Med (Dubai).

[28] Al-Omari MA, Al-Dwairi ZN (2005). Compliance with infection control programs in private dental clinics in Jordan. J Dent Educ.

[29] Huang J, Gan Y, Xu H, Li N, An N, Cai Z (2023). Prevalence and characteristics of needlestick injuries among dental interns during their first-year clinical training: an observational study. BMC Oral Health.

[30] Silva MMS, Nichiata LYI, Simão NS, Silveira RAD (2021). Conditions associated with adherence to HIV post-sexual exposure prophylaxis. Rev Esc Enferm USP.

